# Reviews

**Published:** 1862-12

**Authors:** 


### REVIEWS.

*Health: its Friends and its Foes; by* R. D. Mussey, M. D., LL. D., *late Professor of Anatomy and Surgery at Dartmouth College, N. H., and of Surgery in the Medical College of Ohio; Fellow of the American Academy of Arts and Sciences, etc., etc. Boston:* Gould & Lincoln, 1862.

It appears from the preface of this book that more than thirty years since Dr. Mussey began to take a special interest in the subject of Hygiene. His professional intercourse with different classes of men, in private and hospital practice at home, as well as observations in extensive sanitary institutions abroad, brought under his notice so much suffering from what he regarded as errors in *diet*, *regimen* and *medication*, that he adopted the practice of making notes of facts and cases, and preserving them for future examination, with the hope that, at some time, he should be able to put them in such form as to justify his presenting them to the public, and thus discharge an obligation to his profession, and at the same time do something to promote the welfare of his fellow men. The facts and cases thus gathered form the basis of this volume. While it has been his object to meet the comprehension of the general reader, he has at the same time endeavored to present suggestions which the physician would not regard as beneath his notice. Many truthful and valuable suggestions are made, and yet it must be confessed that the Doctor, learned and impartial as he has generally been considered, has yet some hobbies left. A large number of chapters in this book are devoted to the consideration of the following and similar subjects, and we mistrust that our author establishes his theories by his facts, as fas as possible, but does not always form his theories from their natural and conclusive teachings.

Chapter VIII: Man by nature a vegetable-eater—vegitarianism. Chapter X: Man omniverous by practice—gluttony, sickness and corpulency, &c., &c. Chapter XII: Vegetable food sufficient for man, favorable to health—moral and intellectual effects of a vegetable diet—the Prophet Daniel, &c., &c. He says: "The case of the Prophet Daniel and his companions illustrates some of the foregoing propositions. An experiment of only ten days' duration proved that vegetable food and water promoted both health and beauty; their countenances appeared fairer and fatter in flesh than all the children who did eat the portion of the king's meat."

We must not attempt to quote the argument, but will say that a great deal of persuasion is employed and considerable scripture to convince us that vegetable diet is a much more healthy, moral and religious food than flesh, while those who eat meats are often found to be cruel, inhospitable, brutal and degraded. We shall try to excuse whatever does not seem so conclusive evidence as physicians demand, when we remember the difficulty of making a popular and at the same time a medical book; and yet it seems to us that there is no need of teaching the masses any errors in diet since the popular mind is now overcrowded with most absurd and irrational notions concerning the healthfulness of various articles of food. It seems the principal object of the author to show how very injurious to health is strong drink, narcotics, and flesh-eating. The injurious effects of strong drink and narcotics are sufficiently obvious, but when we come to the beef steak, it would require the whole of the Old and New Testament (which by the way is the fountain of proof in this book) to convince us that a man who eats it, is not as good a Christian as his neighbor, who has been made to believe that "man is by nature a vegetable-eater." This book is written in fine style, and is very entertaining and instructive. By the general reader it will be regarded as exceedingly full of important hints and facts, and well worthy their perusal and consideration.

